# Comparison of an Herbal- and a 0.12% Chlorhexidine-based Oral Rinse as Adjuncts to Nonsurgical Mechanical Debridement in the Management of Peri-implant Mucositis: A Randomised Controlled Trial

**DOI:** 10.3290/j.ohpd.a45069

**Published:** 2020-09-04

**Authors:** Hamad Alzoman, Tariq Ghazi Alojaym, Sandeepa Nuchilakath Chalikkandy, Abid Mehmood, Fayza Rashed, Darshan Devang Divakar

**Affiliations:** a Associate Professor, Department of Periodontics and Community Dentistry, College of Dentistry, King Saud University, Riyadh, Saudi Arabia. Idea, hypothesis, contributed substantially to writing the introduction and discussion, revised the manuscript prior to submission, contributed equally to the present study.; b Dentist, Conservative Dentistry, Asser Specialist Dental Center, Abha, Saudi Arabia. Experimental design, contributed substantially to writing the introduction and discussion, performed the statistical analysis, contributed equally to the present study.; c Assistant Professor, Department of Diagnostic Sciences – Oral Biology, King Khalid University, College of Dentistry, Abha, Saudi Arabia. Experimental design, contributed substantially to writing the introduction and discussion, performed the clinical examinations, contributed equally to the present study.; d Professor, Department of Dentistry, Jinah Hospital, Karachi, Pakistan. Administered the questionnaire, performed the clinical and laboratory-based examinations and wrote the methods, contributed equally to the present study.; e General Dentist, Qassim University, Buraidah, Al-Qassim, Saudi Arabia. Wrote the introduction and discussion and revised the manuscript prior to submission, contributed equally to the present study.; f Researcher, Dental Biomaterials Research Chair, Dental Health Department, College of Applied Medical Sciences, King Saud University, Riyadh, Saudi Arabia. Performed the statistical analysis, wrote the results and revised the manuscript, contributed equally to the present study.

**Keywords:** bleeding on probing, chlorhexidine, herbal, peri-implant mucositis, probing depth

## Abstract

**Purpose:**

In the present randomised controlled trial (RCT), the authors hypothesise that herbal-based oral rinses are as effective as 0.12% chlorhexidine when used as adjuncts to non-surgical mechanical debridement (MD) in the management of peri-implant mucositis. The aim of the present RCT was to compare the efficacy of an herbal oral rinse with a 0.12% chlorhexidine-based oral rinse when used as adjuncts to non-surgical MD in the treatment of peri-implant mucositis.

**Materials and Methods:**

Adult patients with peri-implant mucositis were included. Participants were randomly allocated into 3 groups. In group 1, patients only underwent non-surgical MD. In groups 2 and 3, patients underwent non-surgical MD with adjunct rinsing with an herbal- and 0.12% CHX-based oral rinse, respectively. Peri-implant plaque index (PI) and bleeding-on-probing (BOP) and probing-depth (PD) were assessed at baseline and at 3, 6, and 12 weeks. Group comparisons were done and p < 0.01 was considered statistically significant.

**Results:**

Forty-eight patients with peri-implant mucositis (16, 16, and 16 in groups 1, 2 and 3, respectively) were included. At baseline, there was no difference in PI, PD, BOP in all groups. In group 1, there was no statistically significant difference in PI and BOP at 6, and 12 weeks of follow-up compared with baseline. In groups 2 and 3, PI (p < 0.01) and BOP (p < 0.01) were statistically significantly higher at baseline than 3, 6, and 12 weeks of follow-up. In group 1, there was no statistically significant difference in PD at all time intervals. In groups 2 and 3, PD was statistically significantly higher at baseline than 3, 6, and 12 weeks of follow-up. In groups 2 and 3, there was no statistically significant difference in PI, BOP and PD at all intervals.

**Conclusion:**

Herbal- and 0.12% CHX-based oral rinses are useful adjuncts to MD for the treatment of peri-implant mucositis.

Peri-implant mucositis is characterised by mucosal inflammation, which manifests as bleeding (bleeding on probing [BOP]) on gentle probing of peri-implant margins, and widening of the peri-implant sulci leading to an increased probing depth (PD).^5,20,34^ However, if adequate preventive and therapeutic measures are not taken, the ongoing inflammatory reaction may aggravate and compromise the peri-implant crestal bone leading to peri-implantitis.^6^ The most common factor linked with the aetiology of peri-implant mucositis is poor routine oral hygiene maintenance.^19^ Accumulation of oral biofilm particularly in the peri-implant submucosal region disrupts the host-microbe homeostasis at the implant-mucosal junction.^19^

The classical management protocol of peri-implant mucositis is mechanical debridement (MD) of the biofilm from the supra- and submucosal peri-implant surfaces.^2,33^ However, adjunct therapies such as prescription of chlorhexidine gluconate (CHX)-based antimicrobial oral rinses are often prescribed to facilitate the healing process after non-surgical MD.^32,33^ According to Solderer et al,^39^ CHX is an essential chemopreventive tool that is helpful in reducing oral biofilm formation and mucosal inflammation after MD of peri-implant surfaces. Despite its advantageous antimicrobial characteristics, CHX may induce allergic reactions in susceptible patients.^14^ Type I hypersensitivity reactions, such as anaphylaxis, have been reported following chlorhexidine application to mucous tissues.^31^ According to Kotsailidi et al,^23^ mucositis and a burning sensation in the mouth are manifested in patients with CHX allergy. However, the precise prevalence of CHX allergy in the general population remains unknown.^31^ Herbal-based oral rinses are composed of a variety of natural compounds including catechins, quercetin, caffeic acid, chlorogenic acid, kaempferol, gallic acid, myricetin, and polyphenols,^25^ which possess anti-inflammatory, antimutagenic, antibacterial, and antioxidant properties.^25^ Furthermore, extracts obtained from herbs including *Salvadora persica,* *Terminalia bellirica,* and *Gossia fragrantissima* have been reported to exhibit anti-inflammatory effects when used as oral rinses. In this regard, herbal-based oral rinses seem to be potential alternatives to the traditional 0.12% CHX-based mouthrinses. The literature contains limited evidence on the impact of herbal oral rinses in terms of reducing oral inflammatory conditions. However, it has been reported that herbal oral rinses exhibit anti-inflammatory properties similar to CHX in terms of reduction in plaque index (PI), gingival index (GI) and reduction in gingival crevicular fluid volume.^13^ A clinico-microbiological study^37^ compared the efficacy of an herbal oral rinse with CHX in relation to reduction in the counts of the pathogenic microbes *Tannerella forsythia, Fusobacterium nucleatum* and *Streptococcus mutans*. Those authors^37^ concluded that herbal oral rinses are as antimicrobially effective as CHX. There are no studies in the literature that have compared the efficacy of herbal- and CHX-based oral rinses as adjuncts to non-surgical MD in the treatment of peri-implant mucositis. In the present randomised controlled trial (RCT), the authors hypothesise that herbal-based oral rinses are as effective as 0.12% chlorhexidine when used as adjuncts to non-surgical MD in the management of peri-implant mucositis.

The aim of the present RCT was to compare the efficacy of an herbal-based oral rinse with 0.12% (CHX) when used as adjuncts to non-surgical MD in the treatment of peri-implant mucositis.

## Materials and Methods

### Ethics Statement

The study was designed, conducted and reported following the Consolidation Standards of Reporting Trials (CONSORT) Statement. The present study was performed following guidelines recognised by the Declaration of Helsinki as revised in 2013 for experimentation involving human patients. Ethical approval was obtained from the ethics committee of Gina Hospital, Karachi, Pakistan (OR/DD/4879-0012). Written and verbal oral hygiene instructions were given to all invited individuals regardless of their decision to either decline or accept the invitation. Withdrawal was inconsequential, and signing the consent form was compulsory.

### Inclusion and Exclusion Criteria

Patients diagnosed with peri-implant mucositis were included. The diagnosis of peri-implant mucositis was based on the Consensus Report of Workgroups of the 2017 World Workshop on the Classification of Periodontal and Peri-Implant Diseases and Conditions.^10^ In summary, peri-implant mucositis was defined as the presence of erythema, BOP, swelling and/or suppuration of the peri-implant sites.^10^ Patients with peri-implantitis (patients that exhibited a mesial and/or distal crestal bone loss of ≥ 3mm on digital bite-wing radiographs), tobacco smokers, smokeless tobacco chewers and patients with existing or a history of periodontitis were not included. Pregnant and/or lactating females and patients with self-reported systemic diseases such as cardiovascular diseases, diabetes mellitus (DM), prediabetes, and renal/hepatic diseases were excluded.

### Questionnaire and Evaluation of Patient Records

Particulars about gender and age, quantity of implants placed per individual, numbers of implants diagnosed with peri-implant mucositis in each participant, and duration of implants in function were collected using a questionnaire. The questionnaire was administered by one trained investigator (Kappa score 0.92). Data regarding implant characteristics (platform switching, surface characteristics, depth of placement [bone level/subcrestal], prosthesis retention [cement or screw-retention], emergence profile [convex or concave]) and reason for tooth extraction prior to implant placement were derived from the patients’ dental records.

### Randomisation and Study Group Allocation

Randomisation was done using a site-specific randomisation assignment sequence generated prior to initiation of the present RCT, and allocation to the study groups was done via block randomisation.^11^ Following the confirmation of patient eligibility, a trained researcher electronically produced a randomisation assignment and notified the principal investigator. The participants were divided into 3 groups as follows: in group 1, non-surgical MD of peri-implant surfaces was performed and patients were advised to rinse with 10 ml of distilled water. In group 2, non-surgical MD of peri-implant surfaces was performed and patients were advised to rinse twice daily with 10 ml of an herbal mouthwash (HiOra, Himalaya Drug; Bengaluru, India) for 2 weeks. The chief constituents of the herbal oral rinse were 5 mg *S. persica*, 10 mg *T. bellirica*, 10 mg *G. fragrantissima* and 0.2 mg *Elettaria cardamomum*.^29^ In group 3, non-surgical MD of peri-implant surfaces was performed and patients were instructed to rinse twice daily for 2 weeks with 10 ml of 0.12% CHX. Therapeutically, patient allocation was concealed by placing the therapeutic regimens in opaque envelopes. The research coordinator was responsible for the randomisation and patient allocation. All patients underwent full-mouth scaling and root planing by a trained and calibrated dental hygienist using sterile curettes (Gracey curettes; UNC15, Hu-Friedy; Chicago, IL, USA). Peri-implant MD was performed using plastic curettes (Implant-Prophy Plastic Dental Instrument System Kit, Henry Schein Dental; New Berlin, WI, USA). Oral hygiene instructions were given to all participants.

### Assessment of Clinical Parameters at Baseline and at 3, 6, and 12 Weeks of Follow-up

In all groups, the outcome measures peri-implant plaque index (PI) and BOP were measured using the protocol reported by Löe.^24^ The peri-implant PD^8^ was measured to the nearest millimeter using a plastic graded probe (UNC15 periodontal probe, Hu-Friedy). The probe was inserted into the buccal and palatal/lingual sulci and gently moved down until resistance was felt; the corresponding PD was then recorded. These measurements were performed on 6 surfaces per implant (midlingual/palatal, distolingual/palatal, mesiolingual/palatal, distobuccal, midbuccal, and mesiobuccal) by a skilled, and standardised examiner (Kappa 0.89) who was blinded to the study groups.

### Statistical Analysis and Sample Size Calculation

Group comparisons were done using a statistical software package (SPSS v 21, SPSS; Chicago, IL, USA). Data normality was assessed using the Kolmogrov-Smirnov test. As study variables exhibited normal distribution, ANOVA and Bonferroni’s post-hoc adjustment were conducted to compare significant differences in PI, BOP, and PD between the groups and 3, 6, and 12 weeks of follow-up. A p-value < 0.01 was considered statistically significant. Power analysis was done considering dental implants with mucositis. It showed that 15 patients per group were needed for a statistical power of 95% to detect a real difference in the mean BOP of 0.8 between treatment groups with a standard deviation of 1.7 and α = 5%.^26^

## Results

### General Characteristics

**Fig 1:**
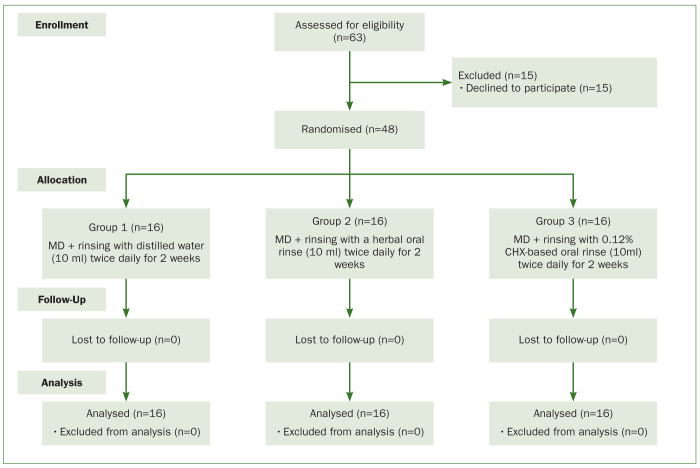
Derivation of the main study groups using the Consolidated Standards of Reporting Trials (CONSORT) flow diagram.

Sixty-three patients with peri-implant mucositis were invited to participate in the present investigation. Fifteen individuals were excluded during the initial screening process as they refused to participate in the present RCT. The derivation of the main study groups and their progress through the stages of the study is shown in the CONSORT flow diagram (Fig 1). Forty-eight patients with peri-implant mucositis (n = 16 each in groups 1, 2, and 3) were included. The gender and mean ages were comparable between patients in these groups. A total of 16 implants were placed in each group. In each group, one dental implant was located in the maxilla or mandible in the region of the missing premolar or molar. In groups 1, 2, and 3, 75%, 87.5%, and 75% of the implants, respectively, were located in the mandible. In groups 1, 2, and 3, the implants were in function for 1.1 ± 0.2, 1.05 ± 0.08, and 1.1 ± 0.1 years, respectively. The diagnosis of peri-implant mucositis in groups 1, 2, and 3 was made 5 ± 1.2, 6 ± 0.2, and 6.3 ± 0.1 days prior to initiation of the present RCT. In all groups, the participants reported brushing their teeth once daily and none of the individuals reported to have ever used a dental floss (Table 1).

**Table 1:** General characteristics of study groups

Parameters	Group 1	Group 2	Group 3
Number of participants	16	16	16
Gender (male:female)	9:7	10:6	10:6
Age (mean ± SD)	41.1 ± 0.6 years	42.4 ± 0.5 years	41.4 ± 0.5 years
Males	42.3 ± 0.4 years	43.2 ± 0.4 years	43.1 ± 0.2 years
Females	39.8 ± 0.2 years	40.4 ± 0.7 years	40.5 ± 0.3 years
Total number of implants	16*	16*	16*
Location (maxilla:mandible)	4:12	2:14	4:12
Number of implants with peri-implant mucositis	16	16	16
Duration of implants in function in years (mean ± SD)	1.1 ± 0.2 years	1.05 ± 0.08 years	1.1 ± 0.1 years
Duration of peri-implant mucositis in days (mean ± SD)	5 ± 1.2 days	6 ± 0.2 days	6.3 ± 0.1 days
Toothbrushing once daily (n)	15	15	15
Daily flossing	None	None	None

The implants were located in the region of a missing premolar or molar.

### Reasons for Tooth Extraction and Implant-related Features

Prior to implant placement, the teeth were extracted for the following reasons: dental caries, endodontic failure, and fractured teeth with embedded root remnants. All implants were platform-switched, had moderately rough surfaces, and were placed at bone level. In all groups, the prostheses were cement retained. All implants had a convex emergence profile.

### Peri-implant Parameters at Baseline and at 3, 6, and 12 weeks of Follow-up

**Fig 2:**
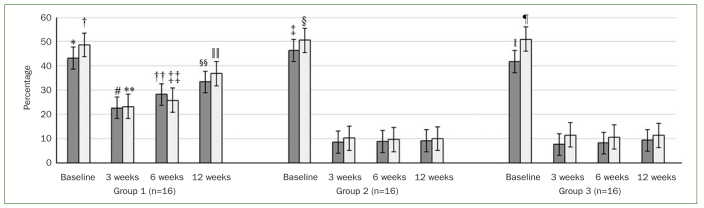
Peri-implant plaque index (PI) (dark grey bars) and bleeding on probing (BOP) (light grey bars) in the study groups at baseline and at 3-, 6-, and 12-week follow-ups. *Compared with PI at 3 weeks of follow-up in group 1 (p < 0.01); †compared with BOP at 3 weeks of follow-up in group 1 (p < 0.01); ‡compared with PI at 3 (p < 0.01), 6 (p < 0.01) and 12 weeks (p < 0.01) of follow-up in group 2; §compared with BOP at 3 (p < 0.01), 6 (p < 0.01) and 12 weeks (p < 0.01) of follow-up in group 2; §compared with PI at 3 (p < 0.01), 6 (p < 0.01) and 12 weeks (p < 0.01) of follow-up in group 3; ¶compared with BOP at 3 (p < 0.01), 6 (p < 0.01) and 12 weeks (p < 0.01) of follow-up in group 3; #compared with PI in groups 2 (p < 0.01) and 3 (p < 0.01) at 3 weeks of follow-up; **compared with BOP in groups 2 (p < 0.01) and 3 (p < 0.01) at 3 weeks of follow-up; ††compared with PI in groups 2 (p < 0.01) and 3 (p < 0.01) at 6 weeks of follow-up; ‡‡compared with BOP in groups 2 (p < 0.01) and 3 (p < 0.01) at 6 weeks of follow-up; §§compared with PI in groups 2 (p < 0.01) and 3 (p < 0.01) at 12 weeks of follow-up; §§compared with BOP in groups 2 (p < 0.01) and 3 (p < 0.01) at 12 weeks of follow-up.

**Fig 3:**
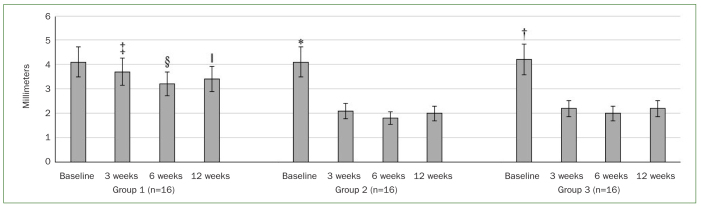
Peri-implant probing depth (PD) in the study groups at baseline and at 3, 6, and 12 weeks of follow-up. *Compared with 3 (p < 0.01), 6 (p < 0.01) and 12 weeks (p < 0.01) of follow-up in group 2; †compared with 3 (p < 0.01), 6 (p < 0.01) and 12 weeks (p < 0.01) of follow-up in group 3; ‡compared with 3 weeks of follow-up in groups 2 (p < 0.01) and 3 (p < 0.01); §compared with 6 weeks of follow-up in groups 2 (p < 0.01) and 3 (p < 0.01); §compared with 12 weeks of follow-up in groups 2 (p < 0.01) and 3 (p < 0.01).

At baseline, there was no statistically significant difference in PI and BOP among patients in groups 1, 2, and 3 (Table 2). At 3 weeks of follow-up, there was a statistically significant reduction in peri-implant PI (p < 0.01) and BOP (p < 0.01) in group 1 compared with baseline. In group 1, there was no statistically significant difference in peri-implant PI and BOP at 6, and 12 weeks of follow-up compared with baseline. In groups 2 and 3, peri-implant PI (p < 0.01) and BOP (p < 0.01) were statistically significantly higher at baseline compared with 3, 6, and 12 weeks of follow-up. At 3, 6, and 12 weeks of follow-up, peri-implant PI (p < 0.01), and BOP (p < 0.01) were statistically significantly higher in group 1 than in groups 2 and 3. There was no statistically significant difference in peri-implant PI and BOP in groups 2 and 3 at 3, 6, and 12 weeks of follow-up (Fig 2). At baseline, there was no statistically significant difference in PD in all groups. In group 1, there was no statistically significant difference in the PD at any time interval. In groups 2 and 3, the peri-implant PD was statistically significantly higher at baseline compared with 3, 6, and 12 weeks of follow-up. In group 1, peri-implant PD was significantly higher at 3 (p < 0.01), 6 (p < 0.01). and 12 weeks (p < 0.01) of follow-up compared with PD at 3, 6, and 12 weeks of follow-up in groups 2 and 3. There was no statistically significant difference in the PD at 3, 6, and 12 weeks of follow-up among patients in groups 2 and 3 (Figs 2 and 3).

**Table 2:** Peri-implant parameters at baseline in the study groups

Baseline parameters	Group 1	Group 2	Group 3
Plaque index (%) (mean ± SD)	43.2 ± 4.5%	46.4 ± 2.7%	41.8 ± 1.6%
Bleeding on probing (%) (mean ± SD)	48.7 ± 1.3%	50.6 ± 0.4%	51.1 ± 0.5%
Probing depth in mm (mean ± SD)	4.1 ± 0.3	4.1 ± 0.2	4.2 ± 0.3

## Discussion

There are no studies that have compared the effectiveness of herbal and CHX-based oral rinses for treatment of peri-implant mucositis. It was not our intention to prove that herbal oral rinses are superior to CHX or vise-versa in terms of their anti-inflammatory efficacy. In the present study, none of the patients reported having or developing CHX allergy; however, it has been reported that CHX may induce type I hypersensitivity reactions.^27,31^ Adverse effects of chlorhexidine-containing oral hygiene products encompass alterations in taste sensation, burning sensation in the mouth, discolourations of restorations, teeth, and/or the ventral tongue surfaces, as well as increased calculus formation.^23,30,31^ In this context, an oral rinse containing a substitute for CHX may most likely be needed, particularly for patients with peri-implant mucositis and CHX allergy. In this regard, the authors of the present RCT hypothesised that there is no difference in the anti-inflammatory effectiveness of herbal and CHX-based oral rinses in terms of reduction in peri-implant soft tissue inflammatory parameters (PI, BOP, and PD). Results of the present RCT are in accordance with the proposed hypothesis, as 3-, 6-, and 23-week follow-up results showed no statistically significant difference between CHX and herbal-based oral rinses in terms of reduction in PI, BOP, and PD. However, both oral rinses caused a statistically significant reduction in the aforementioned inflammatory parameters in contrast with their respective baseline values.

The herbal oral rinse used in the present RCT contains a variety of herbal ingredients, including *S. persica, T. bellirica, G. fragrantissima,* and *E. cardamomum*.^29^ It has been reported that *S. persica* and *T. bellirica* exhibit anti-plaque formation characteristics and have an antimicrobial effect against a variety of microbes including but not limited to lactobacilli, Pseudomonas species, streptococci and *Candida albicans*.^16,17,35^ According to Sofrata et al,^38^ the principal anti-bacterial constituent of *S. persica* is benzyl isothiocyanate, which has a strong bactericidal effect against periodontopathogens, including *Porphyromonas gingivalis* and *Aggregatibacter actinomycetemcomitans.* It is noteworthy that microbes such as *P. gingivalis,* *A. actinomycetemcomitans,* and *C. albicans* have also been associated with the aetiopathogenesis of peri-implant diseases.^4,7,36^ In an RCT by Parwani et al,^28^ the authors compared scores of plaque and gingival indices among patients with gingival inflammation 4 days after SRP and prescription of either an herbal or CHX-based oral rinse. Their results showed that both CHX and herbal oral rinses significantly reduced the gingival and plaque index scores in the patient population under investigation.^28^ It is worth mentioning that the reduction in PI and BOP persisted up to 12 weeks of follow-up in the present study. This suggests that both herbal and CHX-based oral rinses inhibit the growth of oral biofilm and that the plaque inhibitory effect lasts for at least 3 months.

There are, however, a number of limitations of the present study. First, data related to the pre- and post-operative keratinised tissue width (KTW) was unavailable. It has been reported that KTW plays a role in the maintenance of peri-implant health,^15^ and deficiency in KTW is associated with peri-implant soft-tissue recession, loss of attachment, and increased depth of angular bony defects.^15^ According to Buyukozdemir Askin et al,^12^ implants with narrow keratinised mucosa (≤ 2 mm) demonstrate a higher PI compared to peri-implant sites with keratinised mucosa > 2 mm. There is a likelihood that MD with adjuvant CHX or herbal-based oral rinses helps restore KTW; however, the quantity of oral rinse reaching the submucosal zone was not assessed in this study. Another limitation is that MD was solely performed using plastic curettes. It is hypothesised that the use of additional instruments such as ultrasonic scalers could have contributed to reducing the peri-implant clinical inflammatory parameters. In the present RCT, tobacco smokers and immunodeficient patients (e.g. patients with diabetes mellitus) were excluded. Such factors are known to enhance the risk of peri-implant diseases (peri-implant mucositis and peri-implantitis) in susceptible populations.^1,3,21,22^ It is therefore anticipated that the outcomes of MD with adjunct oral rinse therapy (herbal or CHX-based) is compromised in tobacco-product users and immunocompromised patients. Moreover, since the present study focused on the treatment of peri-implant mucositis, the probable benefits of MD with adjunct herbal-based oral rinse in the treatment of peri-implantitis have yet to be determined. Furthermore, in the present RCT, CHX was used at a concentration of 0.12%. There is evidence that CHX at a concentration of 0.2% has a significantly better effect in preventing oral biofilm than at a concentration of 0.12%.^18^ Nevertheless, according to Berchier et al,^9^ CHX – when used at a concentration of either 0.12% or 0.2% – offers negligible benefits in a clinical scenario. This warrants further well-designed, double-blinded and power-adjusted clinical and immunobiological studies with long-term follow-up.

## Conclusion

Herbal- and 0.12% CHX-based oral rinses are useful adjuncts to MD for the treatment of peri-implant mucositis.
